# The Diagnosis and Therapeutic Management of Anomalous Aortic Origin of the Coronary Artery: A Retrospective Study Conducted at a Single Center in China

**DOI:** 10.31083/RCM33432

**Published:** 2025-07-29

**Authors:** Cheng Zhang, Dan Shi, Xiaonan He

**Affiliations:** ^1^Department of Cardiology, China-Japan Union Hospital of Jilin University, 130033 Changchun, Jilin, China; ^2^Department of Radiotherapy, China-Japan Union Hospital of Jilin University, 130033 Changchun, Jilin, China; ^3^Emergency Critical Care Center, Beijing Anzhen Hospital, Capital Medical University, 100029 Beijing, China

**Keywords:** anomalous coronary artery, echocardiography, surgery

## Abstract

**Background:**

This study collected data on the incidence and management of anomalous aortic origin of the coronary artery (AAOCA). We described the incidence of AAOCA and the observed outcomes after management.

**Methods:**

This retrospective study focused on patients treated for AAOCA in a tertiary hospital during the last 20 years. Patients were divided into the anomalous left coronary artery from the pulmonary artery (ALCAPA) group, the non-ALCAPA group, and the symptomatic and asymptomatic groups. Clinical manifestations and related data after surgery were compared among the different groups.

**Results:**

From April 2003 to July 2022, 102 patients were diagnosed with AAOCA and treated at Beijing Anzhen Hospital. ALCAPA was identified as the most prevalent anomaly. The incidence of syncope and heart failure was significantly lower and higher, respectively, in the ALCAPA group. Surgical intervention yielded immediate benefits not only for ALCAPA patients but also for patients who underwent AAOCA. In total, 64.7% of the patients underwent coronary artery osteoplasty, which provided a comprehensive surgical approach addressing all anatomical issues associated with AAOCA. Compared to preoperative measurements, there was a significant reduction in the left ventricular end-diastolic diameter (LVEDD) after surgical intervention (*p* < 0.001). Both the ejection fraction (EF) before and after surgery and the incidence of inter-arterial abnormal vessels in the asymptomatic group were significantly higher than those observed in the symptomatic group (*p* < 0.001).

**Conclusions:**

ALCAPA is most frequently observed among patients with AAOCA. Thus, surgical intervention benefits AAOCA patients, particularly asymptomatic individuals.

## 1. Introduction

The incidence of anomalous aortic origin of the coronary artery (AAOCA) in clinical practice is relatively low, ranging from about 0.1% to 0.9% [1, 2, 3]. This condition primarily affects the pediatric and adolescent populations. Sudden cardiac death (SCD) accounts for about 15–20% of mortality in children and adolescents with AAOCA [4, 5]. While most patients remain asymptomatic, typical clinical manifestations may include angina pectoris, chest pain, palpitations, dyspnea, dizziness, syncope, myocardial infarction, and SCD [1, 3].

The diagnostic management strategy for AAOCA involves the application of transthoracic echocardiography (TTE), coronary computed tomography angiography (CCTA), cardiac magnetic resonance imaging (CMR), and coronary angiography (CAG) [6, 7, 8]. In individuals under 30 years of age, particularly pediatric patients, TTE should be considered the primary noninvasive diagnostic modality because of its safety and absence of exposure to radiation [9, 10]. If definitive exclusion of AAOCA cannot be achieved or if additional imaging is required to evaluate high-risk characteristics [11], CCTA or CMR imaging techniques may be considered [6, 7, 8]. Conversely, for individuals above 30 years of age, direct CCTA is recommended for assessing AAOCA [12].

Surgery is the primary treatment modality for these patients, necessitating a comprehensive surgical strategy due to the pathological mechanisms induced by the AAOCA from the opposite sinus, coronary artery disease, and congenital heart defects. A multicenter study conducted by 45 North American centers affiliated with the Congenital Heart Surgeons’ Society (CHSS) revealed that isolated unroofing was the predominant repair approach (87%), followed by unroofing with commissural manipulation (25%), patch ostioplasty (6%), reimplantation (6%), pulmonary artery translocation (6%), and other strategies [13]. Coronary unroofing is widely used as the predominant surgical technique; however, established guidelines regarding surgical approaches are lacking [14, 15]. Specialized and experienced medical centers are recommended for managing such complex cases. Different surgical protocols should be evaluated based on the anatomical characteristics of each patient.

The available data on the AAOCA in China primarily consists of isolated case reports. In this article, we presented a comprehensive retrospective analysis of AAOCA cases spanning two decades at Beijing Anzhen Hospital, Capital Medical University, which is currently recognized as the largest cardiovascular center in China. This highlights our expertise in diagnosis and treatment, thereby reflecting surgical management outcomes among Chinese patients with AAOCA.

## 2. Materials and Methods

### 2.1 Clinical Data

The present retrospective study included a cohort of 102 patients who were diagnosed with AAOCA and whose data were collected from April 2003 to July 2022 at Beijing Anzhen Hospital, Capital Medical University. Relevant clinical data, including essential demographic information such as sex and age; blood pressure measurements (systolic and diastolic); pulse rates; occurrence of sudden death outside the hospital setting; in-hospital events, including syncope, heart failure, myocardial infarction, and mortality rates; utilization of adjunctive cardiac support devices such as extracorporeal membrane oxygenation (ECMO) or intra-aortic balloon pump (IABP); and imaging research findings, including echocardiography (ECHO), coronary angiography (CAG), and coronary computed tomography angiography (CCTA), were extracted from the hospital database.

In this study, only patients with AAOCA were included while patients with congenital heart diseases, such as atrial or septal defects and patent ductus arteriosus, were excluded. ECHO was performed to assess the ejection fraction (EF) and left ventricular end-diastolic diameter (LVEDD) before and after surgery. The AAOCA patients were categorized into two groups: anomalous left coronary artery from the pulmonary artery (ALCAPA) patients and non-ALCAPA patients. The pertinent information on the two groups of patients was collected, including major clinical manifestation data such as syncope, heart failure, myocardial infarction, and incidence of hospital death, as well as diagnostic methods such as ECHO, CCTA, and CAG. Comparisons of EF and LVEDD on ECHO were conducted before and after surgery for the two groups. Additionally, based on the symptoms of the AAOCA patients, they were categorized into two other groups: symptomatic or asymptomatic. Comparisons between these two groups were made in terms of clinical manifestation data. Some data could not be obtained due to limitations in the database system.

### 2.2 Echocardiography (ECHO)

The ECHO procedure was performed using a Philips IE33 (Best, Eindhoven Metropolitan Area, Netherlands), EPIC 7C color Doppler ultrasound diagnostic instrument equipped with an S5-1 probe operating at a frequency range of 1.5–3.5 MHz. Cardiac morphology and valve regurgitation were comprehensively assessed, encompassing measurements of EF and LVEDD recorded preoperatively and postoperatively.

### 2.3 Coronary Computed Tomography Angiography (CCTA)

The Siemens Somation Definition Flash dual-source computed tomography (CT) scanner was used for CCTA. For adult patients, the examination encompassed the region from 10 mm below the tracheal bifurcation to the level of the diaphragm, whereas for pediatric patients, it extended from the chest entrance to the diaphragmatic level. Prospective electrocardiographic gating technology was used for image acquisition. The nonionic contrast agents Euro Naipaike (350 mg I/mL) or Weishi Paike (320 mg I/mL) were intravenously administered at the elbow via injection. The scan was triggered by automatic tracking technology synchronized with the administration of the contrast agent.

### 2.4 Coronary Angiography (CAG)

CAG procedures were conducted using a standard technique to administer coronary contrast agents, followed by the completion of angiography and simultaneous image recording.

### 2.5 Surgical Techniques

In our institution, surgeons consistently apply personalized surgical techniques with favorable outcomes. The choice of surgical technique was left to the discretion of the surgeon based on the individual anatomy and clinical status.

### 2.6 Statistical Analyses

Statistical analyses were conducted using the SPSS 20 software (SPSS, Chicago, IL, USA). The normality test was performed using the Kolmogorov-Smirnov test, and the results demonstrated that the data follows a normal distribution. Quantitative variables are reported as the mean ± standard deviation, whereas categorical variables are presented as percentages. Student’s paired or unpaired *t*-tests and nonparametric ANOVA (Mann-Whitney test) were performed to determine the differences in continuous variables between groups. The differences between dichotomous variables were determined by the χ^2^ test or Fisher’s exact test when appropriate. All differences between groups were considered to be statistically significant at *p* < 0.05.

## 3. Results

### 3.1 Basic Case Information

Between April 2003 and July 2022, 102 patients were diagnosed with AAOCA, including 49 males (48%) and 53 females (52%). Among these cases, 81 cases originated from the pulmonary artery (76 with involvement of the left coronary artery, four with involvement of the right coronary artery, and one with involvement of both coronary arteries), 12 cases with the right coronary artery (RCA) arising from the left sinus, eight cases from the left coronary artery arising from the right sinus, and one case involving an aberrant connection between the left and right coronary arteries. The mean age at diagnosis was 9.39 ± 14.28 years (range: 28 days–61 years; median: two years). Adults accounted for about 14.7% of all cases, while the rest of the study subjects were minors. The syncope rate among these patients was estimated to be about 7.8% (8/94), whereas the heart failure rate was 50.9% (52/102). Myocardial infarction occurred at a rate of about 5.9% (6/101), whereas sudden death rate was close to 2% (2/100). Additionally, ventricular aneurysms were detected in about 6.8% of patients (7/102), whereas asymptomatic patients accounted for about 38.2% (39/102) (Table 1).

**Table 1.** **Clinical manifestations before surgey**.

Syndromes	Cases	Rate (%)
Syncope	8	7.8
Heart failure	52	50.9
Myocardial infarction	6	5.9
Sudden death	2	2.0
Ventricular aneurysm	7	6.8
Asymptomatic	39	38.2

### 3.2 Clinical Information of the Patients

#### 3.2.1 Diagnostic Methods

The diagnostic methods used were as follows: 81 ECHO (79.4%), nine CAG (8.8%), and 51 CCTA (50%). Diagnosis was performed using a single method in 65 patients (63.7%), two methods in 35 patients (34.3%), and three methods in two patients (2.0%) (Table 2a and 2b).

**Table 2a.** **Preoperative diagnostic methods**.

Methods	Cases	Rate (%)
ECHO	81	79.4
CAG	9	8.8
CCTA	51	50.0

ECHO, echocardiography; CAG, coronary angiography; CCTA, coronary computed tomography angiography.

**Table 2b.** **Number of diagnostic modalities employed before surgery**.

Numbers	Cases	Rate (%)
One method	65	63.7
Two methods	35	34.3
Three methods	2	2.0

#### 3.2.2 Surgery-Related Information

The surgical procedures used in the cases included the correction of coronary artery abnormalities, pedicled left coronary artery transplantation, and coronary artery ostioplasty. A total of 10 patients (9.8%) underwent correction for coronary artery abnormalities, whereas 26 patients (25.5%) underwent pedicled left coronary artery transplantation. Coronary artery ostioplasty was performed on 66 patients (64.7%). The average duration of surgery was 4.5 ± 1.25 h (range: 2.5–10 h; median: 4 h). ECMO was required for six patients (5.9%). Nine patients died in the hospital (8.8%) (Table 3a and 3b).

**Table 3a.** **Surgical techniques**.

Surgical techniques	Cases	Rate (%)
Correction of coronary artery abnormalities	10	9.8
Pedicled left coronary artery transplantation	26	25.5
Coronary artery ostioplasty	66	64.7

**Table 3b.** **Conditions after surgery**.

Conditions after surgery	Cases	Rate (%)
ECMO	6	5.9
Hospital death	9	8.8

ECMO, extracorporeal membrane oxygenation.

### 3.3 Reassessment of Patients Pre-Surgery and Post-Surgery

Of the total 102 cases examined, data on EF and LVEDD analysis were accessible for 80 cases in the hospital database (Table 4). After surgery, the LVEDD decreased significantly relative to the preoperative level (*p* < 0.001). However, there was no statistical difference in EF between pre-surgery and post-surgery (*p* = 0.374).

**Table 4.** **Comparisons of EF (%) and LVEDD pre-surgery and post-surgery in 80 cases**.

Index	Pre-surgery	Post-surgery	*p *value
EF (%)	57.7 ± 13.89	56.6 ± 14.53	0.374
LVEDD (mm)	42.1 ± 9.92	34.8 ± 9.21	<0.001*

* *p* < 0.05. EF, ejection fraction; LVEDD, left ventricular end-diastolic diameter.

### 3.4 Subgroup Analysis of ALCAPA and Non-ALCAPA

Based on the distribution of case numbers, the patients were classified into two groups: the ALCAPA group (74.5%) and the non-ALCAPA group (25.5%). The subsequent data were attributed to this discrepancy.

#### 3.4.1 Basic Information on the ALCAPA Group

The incidence of syncope in this cohort was about 1.3% (1/76), whereas the prevalence of heart failure was about 63.2% (48/76). Additionally, the rate of myocardial infarction was about 2.6% (2/76), sudden death accounted for about 1.3% (1/76), and ventricular aneurysms accounted for about 9.2% (7/76) (Table 5a).

**Table 5a.** **Major clinical manifestations observed in the ALCAPA cohort**.

Clinical manifestation	Cases	Rate (%)
Syncope	1	1.3
Heart failure	48	63.2
Myocardial infarction	2	2.6
Sudden death	1	1.3
Ventricular aneurysm	7	9.2

ALCAPA, anomalous left coronary artery from the pulmonary artery.

#### 3.4.2 Reevaluation of the ALCAPA Group Pre-Surgery and Post-Surgery

The EF and LVDD measurements were obtained before and after surgery for this cohort, with data available for 61 of 76 cases in the hospital database. The findings are presented in Table 5b. Compared to the pre-surgery values, the post-surgery LVEDD values were significantly lower (*p* < 0.001). Conversely, no significant differences were found in the EF between the pre-surgery and post-surgery periods, indicating its stability (*p* = 0.556).

**Table 5b.** **Comparisons of EF (%) and LVEDD pre- and post-surgery in 61 cases**.

Index	Pre-surgery	Post-surgery	*p* value
EF (%)	54.8 ± 14.40	53.9 ± 15.03	0.556
LVEDD (mm)	41.4 ± 10.47	33.7 ± 9.44	<0.001*

* *p* < 0.05.

#### 3.4.3 Comparative Analysis of Syndromes Between the ALCAPA and Non-ALCAPA Groups

The incidence of syncope in the ALCAPA group was significantly lower than that in the residual group (1.3% vs. 26.9%) (*p* < 0.001). Additionally, the ALCAPA group presented a significantly greater incidence of heart failure than the residual group (63.2% vs. 15.4%) (*p* < 0.001). No significant differences were found in the incidence of myocardial infarction, sudden death, or ventricular aneurysm between the two groups. Detailed comparisons can be found in Table 5c.

**Table 5c.** **Comparisons of syndromes between the ALCAPA and non-ALCAPA**.

Syndromes	ALCAPA	Non-ALCAPA	*p* value
Syncope	1	7	<0.001*
Heart failure	48	4	<0.001*
Myocardial infarction	2	4	0.060
Sudden death	1	1	0.447
Ventricular aneurysm	7	0	0.215

* *p* < 0.05.

### 3.5 Subgroup Analysis of the Symptomatic and Asymptomatic Groups

#### 3.5.1 Comparative Analysis of Pre-Surgical and Post-Surgical Outcomes in Groups With Symptomatic and Asymptomatic Conditions

Among 39 patients in the asymptomatic cohort, complete data on EF and LVEDD were available for analysis from 36 individuals via the hospital database, whereas in the symptomatic cohort comprising 63 patients, corresponding data could be obtained from only 55 patients because of missing values or incomplete records. The comparative findings are summarized in Table 6a. No significant differences in the LVEDD either pre-surgical or post-surgical intervention were identified between these two cohorts (*p* = 0.245, *p* = 0.632). However, a significantly greater improvement in EF before and after surgery was found in those initially classified as asymptomatic than in their counterparts who presented symptoms at baseline (*p* < 0.001, *p* = 0.007).

**Table 6a.** **Comparisons of EF (%) and LVEDD in symptomatic and asymptomatic groups pre- and postsurgery separately**.

Group	Index	Symptomatic	Asymptomatic	*p *value
Pre-surgery	EF (%)	52.7 ± 14.97	63.2 ± 10.15	<0.001*
	LVEDD (mm)	40.9 ± 9.53	43.5 ± 10.26	0.245
Post-surgery	EF (%)	53.3 ± 15.83	61.3 ± 10.32	0.007*
	LVEDD (mm)	34.8 ± 9.00	35.8 ± 9.90	0.632

* *p* < 0.05.

#### 3.5.2 The Prevalence of the Interarterial Course in Abnormal Vessels Among Symptomatic and Asymptomatic Patients

The asymptomatic group included 12 patients with an abnormal interarterial course of coronary vessels, resulting in an incidence of 30.8% (12/39). In the symptomatic group, three patients had abnormal interarterial vessels, corresponding to an incidence of 4.8% (3/63). The incidence was significantly greater in the asymptomatic group than in the symptomatic group (*p* = 0.000). Detailed comparisons are presented in Table 6b.

**Table 6b.** **The incidence of the interarterial course for abnormal vessels in the symptomatic and asymptomatic groups**.

	Yes	No	Rate (%)
Symptomatic	3	60	4.8
Asymptomatic	12	27	30.8
*p* value	<0.001*		

* *p* < 0.05.

## 4. Discussions

Our study represents one of the few detailed presentations of AAOCA patient characteristics in such a large reference population. This is explained by the fact that most of the included patients were initially diagnosed in local hospitals, and referred to our institution because their complex and rare conditions, while more straightforward cases of AAOCA were managed locally. So there is a degree of selection bias in our population towards more complex cases and sicker patients. Based on the available data sources analyzed, several key findings have been identified.

### 4.1 Patient Characteristics

Within the patient cohort examined in this study, a slightly greater proportion of female individuals than male individuals were observed. Additionally, most of the included patients in the sample were pediatric patients. The prevailing clinical presentations included heart failure, syncope episodes, myocardial infarctions, ventricular aneurysms, and cases of SCD, with most patients exhibiting one to several of these clinical presentations. Heart failure was the most commonly encountered syndrome among all cases. A subgroup of asymptomatic individuals (38.2%) did not manifest any of the aforementioned syndromes but instead reported atypical symptoms such as fever, dyspnea, or chest pain. These unique cases were sporadically identified in local healthcare facilities.

### 4.2 Diagnostic Methods

In terms of the diagnostic methods used, ECHO was the most widely used noninvasive and cost-effective technique without the use of radiation. In this study, children constituted about 85.3% of the sample population, highlighting the advantages of ECHO for the diagnosis of pediatric cases. A study has reported that AAOCA can be reliably identified with intramural courses via ECHO [3]. Consequently, the proportion of ECHO diagnoses was also relatively high in this context. CCTA, as an alternative noninvasive modality, shows superior applicability (50%). These two primary approaches are commonly and routinely used because of their noninvasive characteristics, particularly when dealing with pediatric patients. Therefore, they serve as primary diagnostic methods that effectively optimize medical resource utilization.

Most patients (63.7%) could be diagnosed using a single method, whereas 34.3% required two methods for diagnosis. Noninvasive techniques are extensively used in clinical practice because of their convenience and cost-effectiveness. ECHO provides a convenient and cost-effective diagnostic approach; however, it cannot detect myocardial infarction [16]. Hence, there is a consistent need for supplementary diagnostic modalities. Intravascular ultrasound (IVUS), through simulated methodologies, facilitates the reliable assessment of myocardial infarction [17]. However, its high cost restricts its routine application in China. In this study, CCTA and CAG were integrated with other diagnostic approaches to perform accurate diagnosis.

### 4.3 Subgroup Analysis

In the general population, the prevalence of ALCAPA is about 0.021% [18]. Within our cohort of 102 patients, ALCAPA accounted for about 74.5%, which was similar to the findings of previous studies. The prevalence of heart failure was about 63.2%. Considerable differences were found in the incidence of syncope and heart failure among ALCAPA patients; The incidence of syncope was low, while the incidence of heart failure was high. These findings suggested that patients with ALCAPA face a greater risk of developing heart failure than experiencing syncope. Because the left coronary artery arises from the pulmonary artery instead of the aorta. After birth, as pulmonary artery pressure drops, the left coronary artery receives deoxygenated blood, leading to myocardial ischemia. This impairs left ventricular function, causing progressive heart muscle damage and eventual heart failure. Syncope in ALCAPA is less common and may arise from acute ischemia-induced arrhythmias or sudden drops in cardiac output. The syncope risk here likely stems from secondary arrhythmias.

In this study, 38.2% of the total population consisted of asymptomatic patients. The incidence of coronary anomalies with an interarterial course was significantly greater in asymptomatic patients (30.8%) than in symptomatic patients (4.8%), suggesting a greater prevalence among those without symptoms.

### 4.4 Surgical Techniques and Results

Regarding surgical techniques, specific recommendations are available for each approach, with an emphasis on their distinctive characteristics [12]. Although the coronary “unroofing” technique is commonly used for AAOCA [19], our findings indicated limited application of this technique due to the presence of these symptom-free individuals. Instead, this study cohort predominantly underwent procedures such as coronary artery ostioplasty and pedicled left coronary artery transplantation as patients candidates for simpler surgical techniques like unroofing were often treated in local hospitals and therefore nor referred to our institution for treatment and inclusion in the present analysis. Surgeries such as coronary “unroofing” were feasible; however, more complex procedures were necessary under specific circumstances.

In this study, coronary artery ostioplasty was performed in 66 patients (64.7%), making it the most frequently used surgical approach for ALCAPA because it can address all anatomical issues directly. However, this procedure presents challenges requiring expertise and specialized nursing care [20, 21].

After surgical intervention, the LVEDD decreased significantly compared to the baseline condition before surgery; however, no substantial change in the EF was noted after surgery. This indicated that significant changes in post-surgery cardiac function was combined with a positive outcome resulting from surgical intervention, indicating significant changes in post-surgery cardiac function, a positive outcome resulting from surgical intervention on cardiac functionality.

Before and after surgery, asymptomatic patients consistently presented higher EF values than symptomatic patients. However, no significant differences in the LVEDD were observed between the two groups. A retrospective study conducted over 20 years in France revealed that about 25% of asymptomatic patients remain asymptomatic, 65% of patients are at risk of coronary artery abnormalities, and 38% of patients experience interatrial and/or intramural courses of coronary artery anomalies [16]. Considering the risks associated with interatrial abnormalities such as SCD and severe events, timely diagnosis and surgical intervention are crucial for these individuals. Hence, unlike symptomatic patients, asymptomatic individuals do not exhibit specific syndromes but are more prone to interatrial and/or intramural courses of coronary artery anomalies. Appropriate surgical interventions need to be implemented to mitigate the risk of SCD.

## 5. Limitations

In this retrospective study, as data were obtained from a single center, information from local hospitals could not be available. Logistical constraints arising from patients’ diverse origins across provinces and districts resulted in unavailable follow-up data for assessing overall survival rates and postsurgical side effects upon their return to their hometowns. As analysed earlier there is a degree of selection bias in our population towards more complex cases and sicker patients. Therefore, these patients may not fully represent the overall landscape of AAOCA cases in China. Thus, some statistical analyses probably failed to provide a comprehensive overview of the AAOCA in China. To address this issue, future studies should prioritize the establishment of multicenter studies.

There could be an underestimation of the prevalence of AAOCA because patients were diagnosed if they had symptoms or if they had examinations for other reasons and systemic screening of all the population is not feasible. Hence, certain asymptomatic AAOCA cases are undetected.

## 6. Conclusions

Although AAOCA is rare in clinical practice, few patients in China present with typical syndromes. Among these cases, ALCAPA is the most frequently observed condition. Surgical intervention not only has immediate effects on ALCAPA patients but is also beneficial for AAOCA patients, particularly asymptomatic individuals, because of the high prevalence of interatrial anatomical abnormalities.

## Data Availability

All data points generated or analyzed during this study are included in this article and there are no further underlying data necessary to reproduce the results.

## Abbreviations

AAOCA, anomalous aortic origin of the coronary artery; ALCAPA, anomalous left coronary artery from the pulmonary artery; LVEDD, left ventricular end-diastolic diameter; EF, ejection fraction; SCD, sudden cardiac death; TTE, transthoracic echocardiography; CCTA, coronary computed tomography angiography; CMR, cardiac magnetic resonance imaging; CAG, coronary angiography; CHSS, Congenital Heart Surgeons’ Society; ECMO, extracorporeal membrane oxygenation; IABP, intra-aortic balloon pump; ECHO, echocardiography.
